# Assessment of the ability of CT urography with low-dose multi-phasic excretory phases for opacification of the urinary system

**DOI:** 10.1371/journal.pone.0174800

**Published:** 2017-04-06

**Authors:** Hiroshi Juri, Takahiro Tsuboyama, Mitsuhiro Koyama, Kiyohito Yamamoto, Go Nakai, Atsushi Nakamoto, Kazuhiro Yamamoto, Haruhito Azuma, Yoshifumi Narumi

**Affiliations:** 1 Department of Radiology, Osaka Medical College, 2–7, Daigaku-machi, Takatsuki, Osaka, Japan; 2 Department of Radiology, Osaka University School of Medicine, 2–15 Yamadaoka, Suita, Osaka, Japan; 3 Department of Urology, Osaka Medical College, 2–7, Daigaku-machi, Takatsuki, Osaka, Japan; Northwestern University Feinberg School of Medicine, UNITED STATES

## Abstract

**Objective:**

To prospectively evaluate the ability of CT urography with a low-dose multi-phasic excretory phase for opacification of the urinary system.

**Materials and methods:**

Thirty-two patients underwent CT urography with low-dose multi-phasic s using adaptive iterative dose reduction 3D acquired at 5-, 10-, and 15-minute delays. Opacification scores of the upper urinary tracts and the urinary bladder were assigned for each excretory phase by two radiologists, who recorded whether adequate (>75%) or complete (100%) opacification of the upper urinary tract and urinary bladder was achieved in each patient. Adequate and complete opacification rates of the upper urinary tracts and the urinary bladder were compared among three excretory phases and among combined multi-phasic excretory phases using Cochran's Q test.

**Results:**

There was no significant difference among three excretory phases with 5-, 10-, and 15-minute delays in adequate (56.3, 43.8, and 63.5%, respectively; P = 0.174) and complete opacification rates (9.3, 15.6, and 18.7%, respectively; P = 0.417) of the upper urinary tracts. Combined tri-phasic excretory phases significantly improved adequate and complete opacification rates to 84.4% and 43.8%, respectively (P = 0.002). In contrast, there were significant differences among three excretory phases for the rate of adequate (31.3, 84.4, and 93.8%, respectively; P<0.001) and complete opacification (21.9, 53.1, and 81.3%, respectively; P<0.001) of the urinary bladder. Multi-phasic excretory phases did not improve these rates because opacification was always better with a longer delay.

**Conclusion:**

Although multi-phasic acquisition of excretory phases is effective at improving opacification of the upper urinary tracts, complete opacification is difficult even with tri-phasic acquisition.

## Introduction

CT urography (CTU) is an excellent technique for the evaluation of calculi and masses in the urinary system [1–4]. Moreover, according to the guideline of European Society of Urogenital Radiology (ESUR), CTU is recommended as the first-line test for patients at high risk of urothelial carcinoma [5], and for these patients, a three-phase CTU protocol with single-bolus contrast material injection consisting of unenhanced, urothelial, and excretory phases is commonly used [5]. The primary goal of the excretory phase (EP) in CTU is complete opacification of the entire urinary system, but incomplete opacification of the urinary system on the EP has been a problem with CTU due to the characteristic points of narrowing and peristalsis. Researchers have suggested several techniques to improve opacification of the upper urinary tracts [6–11], including longer delay for the EP, acquisition of bi-phasic EPs, oral hydration, intravenous diuretic, and abdominal compression. A log-rolling procedure prior to the EP scans was suggested to increase the percentage of bladder opacification [12]. These previous studies assessed the opacification of each segment of the urinary systems separately. Therefore, the ability of CTU to achieve complete opacification of the entire urinary system of patients has not been clearly described, and optimal delay for opacification of the urinary system has not yet been standardized.

Recently, dose reduction has been made possible without any degeneration of image quality by using iterative reconstruction algorithms [13, 14]. These algorithms are post-processing procedure that reducing image noise and artifact as a result of using low radiation dose. One of these algorithms, adaptive iterative dose reduction 3D (AIDR 3D), was recently shown to permit CTU with a diagnostic efficacy comparable to that of routine-dose CTU with filter-back projection (FBP) but with an approximately 70% dose reduction [15]. Therefore, we hypothesized that tri-phasic low-dose EPs using AIDR 3D may be available without increasing the radiation dose and may be effective in achieving complete opacification of the urinary system. To our knowledge, this is the first report to evaluate tri-phasic EP scans.

The purpose of this study was to prospectively compare opacification of the urinary system among 5-, 10-, and 15-minute delays, and to evaluate whether multi-phasic acquisition could achieve complete opacification of the upper urinary tracts on EP CTU.

## Materials and methods

Our institutional review board (Full name: Ethnics Committee of Osaka Medical College) approved this prospective study. The individual in this manuscript has given written informed consent to publish these case details.

### Patients

Between August 2012 and February 2013, we enrolled 40 consecutive patients who met the following four inclusion criteria: the presence of macroscopic hematuria, post-nephroureterestomy of right or left side, or a follow-up study after preservation therapies for bladder cancer; age over 40 years; absence of contraindications for the use of iodinated contrast material; and a serum glomerular filtration rate of 45 or more. Eight patients were excluded because of hydronephrosis, leaving 32 patients (24 men, 8 women; mean age, 65.1 years; range, 42‒84 years; mean weight, 66.1 kg; range, 46‒90 kg). Four patients had undergone nephroureterectomy of the left side, so we evaluated only the right upper urinary tracts for these patients.

### Multidetector CT urographic technique

We obtained unenhanced, urothelial, and tri-phasic EPs for all patients on a 320-row detector CT scanner (Aquilion ONE; Toshiba Medical Systems Corporation, Tokyo, Japan) at the following settings: rotation time, 0.5 seconds; detector collimation, 64 × 0.5 mm; helical pitch, 53; tube voltage, 120 kV; variable tube current determined by x, y, z-axis dose modulation, adjustable with auto exposure control. The current tube was adjusted to 10‒550 mA. Acquisitions were from the top of the renal cortex to the bottom of the urinary bladder on the EPs. Patients were instructed to hold their breath with tidal inspiration during scanning. Oral hydration and administration of an intravenous diuretic were not used before the examinations. A single-bolus injection of 540 mgI/kg of iohexol (Omnipaque; Daiichi Sankyo, Tokyo, Japan) was made with an injection time of 50 seconds (contrast injection rate: 1.7‒2.3 ml/sec), and then tri-phasic EPs using AIDR 3D were acquired 5, 10, and 15 minutes later. The patients were rolled twice immediately prior to each EP scan for better opacification of the urinary bladder. In the same way with the prior study [15], we chose a setting of 75% reduction ratio of the radiation dose under AIDR 3D on the EPs. The CT data on the EP scans were reconstructed with AIDR 3D with a slice thickness and reconstruction interval of 1.0 mm for the axial images. From the axial images, coronal images with a slice thickness and reconstruction interval of 3.0 mm were obtained. A maximum intensity projection (MIP) image was also obtained for each EP.

### CT image analyses

**Fig 1:**
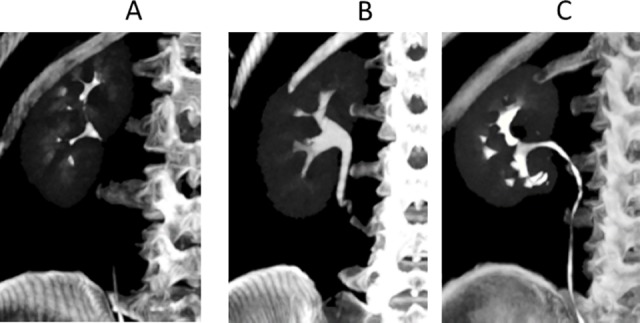
Examples of the evaluation of opacification scores of the proximal ureter on maximum intensity projection (MIP) images. A, A 5-minute delay scored as 1 (<50%); B, A 5-minute delay scored as 2 (51–99%); and C, A 5-minute delay scored as 3 (100%).

Qualitative analysis―Two radiologists with 12 and 7 years of experience in abdominal CT reviewed the EP images using a Picture Archiving and Communication System (PACS) with a preset window level and a window width of 200/600 HU, and performed independent visual evaluations. They were blinded to the patient’s clinical data and to any prior or follow-up imaging results. They evaluated the axial, coronal and MIP images in the three EPs. The upper urinary tracts were classified into 4 segments: the renal collecting system, proximal ureter (the ureteropelvic junction to the iliac crest), middle ureter (the iliac crest to the crossing of the iliac vessels), and distal ureter (the crossing of the iliac vessels to the ureterovesical junction), and each segment on each of the three EPs with 5-, 10-, and 15-minute delays was scored in regard to opacification as follows: 1, <50% opacification; 2, 50–99%; 3, 100% (Fig 1). The urinary bladder was assigned an opacification score for each EP using a 5-point scoring system: 1, <25% opacification; 2, 25‒49%; 3, 50‒74%; 4, 75‒99%; 5, 100% opacification on each EP. Discrepancies in the opacification scores between the two readers were resolved by consensus.

According to the results of the opacification scores, we recorded whether complete and adequate opacification of the upper urinary tracts or the urinary bladder was achieved for each patient on each EP (5-, 10-, and 15-minute delays), and also on combined bi- (combination of 5+10-, 5+15-, and 10+15-minute delay scans), and tri-phasic EPs (combination of 5+10+15-minute delay scans). We defined complete and adequate opacification as 100% and more than 75% opacification, respectively. The upper urinary tracts were considered to have complete opacification if all eight segments of both upper urinary tracts (four segments in patients after nephroureterectomy of the left side) had a score of 3, and adequate opacification if at least three-quarters of all segments had a score of 3. For the urinary bladder, a score of 5 indicated complete opacification while a score of 4 or 5 indicated adequate opacification. In the assessment of multi-phasic EPs, the best score assigned for each segment of the upper urinary tracts and the urinary bladder during the combined multi-phasic EPs was used. Then, the complete and adequate opacification rates of the upper urinary tract and urinary bladder were compared among the three single-phasic acquisitions and among single-, bi- and tri-phasic acquisitions. For comparison among single-, bi-, and tri-phasic acquisitions, we used one single- and bi-phasic EPs with the highest opacification rates among each group. If scores were higher with a longer delay in all patients, we omitted the comparison of multi-phasic EPs.

**Fig 2:**
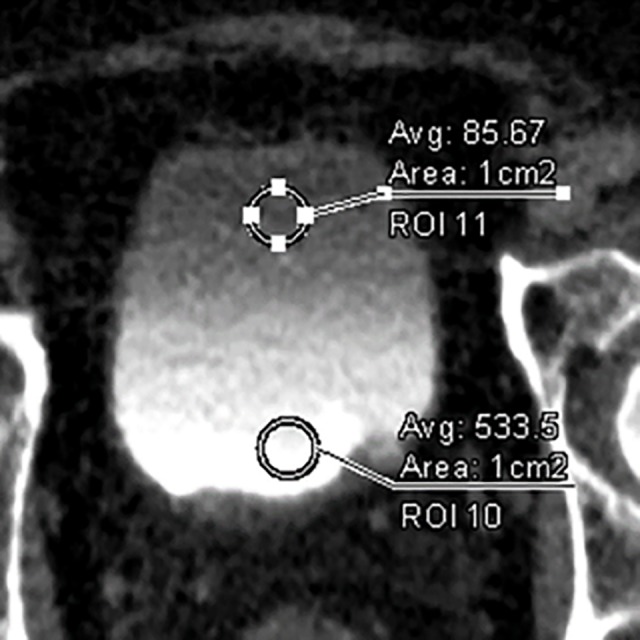
Axial CT images obtained in the excretory phase with a 5-minute delay in a 67-year-old man, with ROIs manually drawn at the anterior and posterior portions of the urinary bladder. The sizes of the ROIs were kept constant on the anterior and posterior portions with a copy-and-paste function.

Quantitative analysis―Homogeneity of the urinary bladder was assessed quantitatively according to the anterior-to-posterior attenuation ratio, which was calculated by dividing the CT value at the anterior portion of the urinary bladder by that at the posterior portion (Fig 2). The CT value was measured with a 100-mm^2^ circular region of interest (ROI) cursor at each portion placed by one radiologist with 8 years of experience in abdominal CT.

### Radiation dose

To estimate the radiation dose on the three EPs, the CT dose index volume (CTDIvol) of each EP was recorded. Then, the average and the total radiation doses on the three EPs were calculated.

### Statistical analysis

Opacification scores were compared using the Friedman test. If there were significant differences among groups, pairwise comparisons were performed with the Wilcoxon signed rank test with Bonferroni correction. To compare the number of patients with complete or 75% opacification of the upper urinary tracts, we used Cochran's Q test and applied the McNemar test with Bonferroni correction to adjust for pairwise comparisons. We used a repeated analysis of variance for quantitative analysis, and applied Tukey’s test to adjust for pairwise comparisons. A value of p < 0.05 was considered to indicate a significant difference for the Friedman test, Cochran's Q test, and repeated analysis of variance. A value of p < 0.0167 was considered to indicate a significant difference for pairwise comparisons. All statistical analyses were performed with SPSS 17.0 (SPSS Inc., Chicago, IL).

## Results

### Qualitative analysis

For opacification scores for the upper urinary tracts, there was no significant difference among three single-phasic EPs in any of the segments (Table 1). On the other hand, there were significant differences in the opacification scores for the urinary bladder among the three EPs (p < 0.001, Table 1). In pairwise comparisons, the opacification scores for the urinary bladder were always significantly higher with a longer delay (5- vs 10-minute delay, p < 0.001; 5- vs 15-minute delay, p < 0.001; 10- vs 15-minute delay, p = 0.002).

**Table 1:** Opacification scores of the urinary systems.

	Delay time on each excretory phase			
	5 minute	10 minute	15 minute	p value
Right				
Renal collecting systems	20/12/0	22/10/0	25/7/0	0.18
Proximal ureter	29/1/2	30/2/0	30/2/0	0.48
Middle ureter	22/8/2	26/4/2	22/8/2	0.49
Distal ureter	17/9/6	19/7/6	15/11/6	0.84
Left				
Renal collecting systems	15/13/0	18/10/0	18/10/0	0.36
Proximal ureter	23/4/1	23/4/1	24/2/2	0.26
Middle ureter	16/8/4	15/9/4	19/6/3	0.51
Distal ureter	15/6/7	15/8/5	15/8/5	0.85
Urinary bladder	7/4/8/9/5	17/10/3/2/0	26/4/2/0/0	<0.001

Footnote: Data show the number of tracts rated as 3, 2, and 1 in each segment on theupper urinary tracts, and show numbers rated as 5, 4, 3, 2, and 1 on the urinary bladder.

The number in the parentheses indicate kappa values.

**Fig 3:**
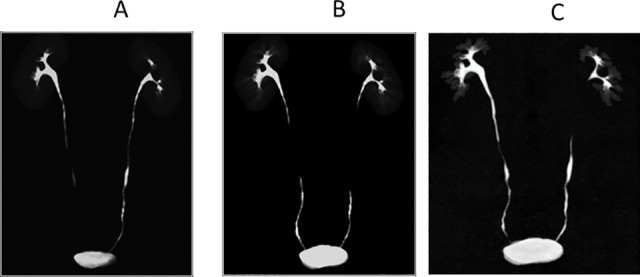
Maximum intensity projection images show the upper urinary tracts obtained in the three excretory phases in a 44-year-old man. A, Excretory phase with a 5-minute delay. B, Excretory phase with a 10-minute delay. C, Excretory phase with a 15-minute delay. With a 5-minute delay, non-opacification was noted in the right ureter, but with a 10- or 15-minute delay, the non-opacified segments were delineated. On the other hand, just a 5-minute delay was sufficient for complete opacification of the left ureter. The numbers of completely opacified segments were 5, 4, 6, 6, 7, 7, and 8 with the 5-, 10-, 15-, 5 + 10-, 5 + 15-, 10 + 15-, and 5 + 10 + 15-minute delays, respectively. Multi-phasic acquisition was thus able to delineate more segments.

The numbers of patients with complete and adequate opacification of the upper urinary tracts in the three obtained EPs are shown in Table 2. Although complete and adequate opacification of the upper urinary tracts were achieved more often on the EP with a 15-minute delay, there was no significant difference among the three EPs (p = 0.417, and 0.174, respectively). Complete opacification was achieved only with a 5- or 10-minute delay in three patients. On bi-phasic EPs, complete opacification of the upper urinary tracts was achieved in 6 (18.7%), 9 (28.1%), and 12 (37.5%) patients with 5+10-, 5+15-, and 10+15-minute delays, respectively, and adequate opacification of the upper urinary tracts was achieved in 26 (81.3%), 26 (81.3%), and 24 (75.0%) patients, respectively. On tri-phasic EPs, 14 (43.8%) and 27 (84.4%) patients showed complete and adequate opacification, respectively. There were significant differences for the rate of complete and adequate opacification among single- (15-minute delay), bi- (10+15-minute delays for complete opacification, 5+15-minute delays for adequate opacification), and tri-phasic acquisitions (5+10+15-minute delays) (p = 0.002 for both, Table 3, Fig 3). In pairwise comparisons, a significant difference in the rate of complete and adequate opacification was found only between single- and tri-phasic acquisition (p = 0.008, and 0.016, respectively).

**Table 2:** Comparison among single-phasic excretory phase for complete and adequate opacification of the upper urinary tracts and the urinary bladder.

	A delay time of the excretory phase			
	5 minute	10 minute	15 minute	p value
Upper urinary tracts				
Complete opacification	3/32 (9.3%)	5/32 (15.6%)	6/32 (18.7%)	0.417
Adequate opacification	18/32 (56.3%)	14/32 (43.8%)	20/32 (63.5%)	0.174
Urinary bladder				
Complete opacification	7/32 (21.9%)	17/32 (53.1%)	26/32 (81.3%)	< 0.001
Adequate opacification	10/32 (31.3%)	27/32 (84.4%)	30/32 (93.8%)	< 0.001

Footnote: Data show the number of patients. Data in parentheses are percentage of patients. Complete and adequate opacification indicate 100% and more than 75% opacification, respectively.

**Table 3:** Comparison among single-, bi-, and tri-phasic excretory phase for complete and adequate opacification of the upper urinary tracts.

	Single-phasic	Bi-phasic	Tri-phasic	p value
Complete opacification	6/32 (18.7%)	12/32 (37.5%)	14/32 (43.8%)	0.002
Adequate opacification	20/32 (63.5%)	26/32 (81.3%)	27/32 (84.4%)	0.002

Footnote; Data show the number of patients. Data in parentheses are percentage of patients. Complete and adequate opacification indicate 100% and more than 75% opacification, respectively. Data of single- and bi-phasic excretory phase are the best results obtained among each group (single-phasic, 15-minute delay; bi-phasic, 10+15-miunute delay for complete opacification and 5+15-minute delay for adequate opacification).

The numbers of patients with complete and adequate opacification of the urinary bladder on the three obtained EPs are shown in Table 2. There were significant differences in the complete and adequate opacification rates among the three EPs (p < 0.001 for both). In pairwise comparisons, EP with a longer delay always showed a significantly higher rate of complete opacification (5- vs 10-minute delay, p = 0.001; 5- vs 15-minute delay, p < 0.001; 10- vs 15-minute delay, p = 0.004). As for adequate opacification, there were significant differences between 5- and 10-minute (p < 0.001), and between 5- and 15-minute delays (p < 0.001). There was no significant difference between 10- and 15-minute delays (p = 0.031). We omitted the evaluation of combined multi-phasic acquisition for opacification of the urinary bladder because the opacification scores were always higher with longer delays.

### Quantitative analysis

The average CT values of anterior and posterior portions of the urinary bladder, and the average anterior-to-posterior attenuation ratios on EPs with 5-, 10-, and 15- minute delays are shown in Table 4.

**Table 4:** Homogeneity of the urinary bladder.

	Mean CT value			p value
	5 minute	10 minute	15 minute	
Anterior portion (HU)	155.1	316.4	493.1	
Posterior portion (HU)	640.0	766.0	816.3	
The ratio of the CT value	0.30	0.47	0.64	<0.001

Footnote: p value is related to the statistical analysis of the ratio of the CT value among three EPs.

There were significant differences in the CT values among the three EPs (Table 4, p < 0.001). In pairwise comparisons, there were significant differences between the 5- and 15-minute delays (p < 0.001). There was no significant difference between the 5- and 10-minute delays (p = 0.037) or the 10- and 15-minute delays (p = 0.044).

### Radiation dose

The average CTDIvols for the 5-, 10-, and 15-minute delay scans were 3.01, 3.04, and 3.01 mGy, respectively. The average CTDIvol was 3.03 mGy on one scanning of the EP. The average total radiation dose on tri-phasic EPs was 9.08 mGy.

## Discussion

Our results demonstrated that longer delay time for EP showed a trend for a higher rate of complete opacification of the upper urinary tracts, and tri-phasic EPs significantly increased the rate of complete opacification. However, complete opacification of the upper urinary tracts was achieved in less than half of the patients even with tri-phasic EPs. Although previous studies have reported complete opacification rates of 37 to 95% for each segment of the upper urinary tract [8, 16], the numbers of patients with entirely opacified upper urinary tracts on CTU have not been reported. Moreover, complete opacification has been defined differently among the prior reports, with Meindle et al. [9] considering more than 75% opacification to be complete, whereas Hack et al. [17] defined complete opacification as 100% opacification. Therefore, we defined complete and adequate opacification as 100% and more than 75% opacification, respectively. To the best of our knowledge, this is the first report to precisely identify the complete opacification rate of the upper urinary tract by CTU.

According to prior reports, EPs have been obtained between 2.5 and 16 minutes after administration of contrast material [4–10, 14–16]. Meindle et al.[8] proposed that a longer delay time of 10 to 16 minutes is feasible for opacification of the distal ureter, and Caoili [10] reported that opacification scores of the distal ureter were statistically improved with a 450-second delay compared to a 300-second delay, which is consistent with our favorable results at a longer delay time of 15 minutes. However, EPs with a 15-minute delay time were not always superior to those with a 5- or 10-minute delay in our study, probably because peristalsis of the ureters can occur regardless of the delay time. It is worth noting that the adequate opacification rates of the upper urinary tract were almost identical among EPs with 5-, 10-, and 15-minute delays, suggesting that a 5-minute delay may be clinically sufficient for adequate opacification of the upper urinary tract even if complete opacification is not achieved.

Both our results and those of a previous study on bi-phasic EPs showed that multi-phasic acquisition of EP can be a good way to increase the likelihood of complete opacification [9]. Multi-phasic EPs can increase the chance of avoiding peristalsis of the ureter because it is difficult to predict when peristalsis will occur. However, there seem to be two problems with multi-phasic EPs. First, complete opacification of the upper urinary tracts is difficult even with tri-phasic EPs, which may limit tumor detection. However, recent studies have called into question the validity of EP for tumor detection, because Metser et al. [16] showed that the detection of urothelial carcinomas was higher on the urothelial phase than on the EP, and Hack et al. [17] demonstrated that tumors at the non-opacified portion of the ureter on EPs were identifiable by other signs on the urothelial phase, such as an enhancing mass or urothelial thickening, and additional multi-phasic EPs for a non-opacified ureter did not improve tumor detection. Accordingly, we need to reconsider whether we really need to aim for complete opacification on CTU because incomplete opacification of the upper urinary tract on EP may not cause any clinical problem. Second, the radiation dose is increased with multiphasic EPs. In the ESUR guideline for CTU, the recommended dose for EP is 9‒12 mGy in CTDIvol for patients at high risk of urothelial carcinoma and 5‒6 mGy in CTDIvol for patients at low or intermediate risk of urothelial carcinoma [5]. In our study, the mean CTDIvol was 3.03 mGy for each EP and 9.08 mGy for the total tri-phasic EP. Even with the low doses that AIDR 3D make possible, the radiation dose for tri-phasic EPs exceeded the recommended dose for patients at low or intermediate risk of urothelial carcinomas.

Considering the drawbacks of multi-phasic EPs and the inconclusive clinical value of complete opacification of the upper urinary tracts for tumor detection, we suggest using single-phase EP as long as it is combined with a urothelial phase scan, even though multi-phasic EPs can improve opacification of the upper urinary tracts. A delay time of longer than 5 minutes may be acceptable for adequate opacification of the ureters on EP.

In this study, opacification scores and the mean anterior-to-posterior attenuation ratios of the urinary bladder were always and significantly higher with a longer delay; hence, there was no inherent usefulness in multi-phasic acquisitions. According to our results, a 10-minute delay seems sufficient for adequate opacification. However, a 15-minute delay seems to be necessary for complete opacification. Theoretically, a much longer delay time may be useful for further improvement of opacification of the urinary bladder. However, there is a controversy regarding the additional value of EP at the urothelial phase for detecting bladder tumors; cystoscopy may be more available than ureteroscopy. Therefore, we consider that a delay time of up to 15 minutes would be feasible for opacification of the urinary bladder.

Our study has some limitations. First, the number of patients was small. Second, we did not use the technique of oral hydration, intravenous diuretic, or abdominal compression because these techniques may be a burden for patients, especially those with chronic heart failure or diabetes. In a prior report, these techniques were useful for better opacification of the upper urinary tract. Therefore, the results in our study may not be applicable to centers where these techniques are used. Third, we did not enroll patients with hydronephrosis. Opacification scores might have been worse if we had enrolled those patients. Finally, we did not use a split-bolus injection technique, in which dual-phase injection of contrast material and one scan for the urothelial and excretory phase is used for the sake of dose reduction. If we had used a split-bolus technique, the volume of contrast material would have been about half that of this study; thus, opacification scores of both the upper urinary tract and the urinary bladder may have been different.

In conclusion, a 15-minute delay on the EP improves opacification of the urinary bladder compared to a 5- or 10-minute delay, but not that of the upper urinary tract.

Although opacification is improved with multi-phasic acquisition, complete opacification is difficult even with tri-phasic acquisition.

## References

[1] Nawfel RD, Judy PF, Schleipman AR, Silverman SG. Patient radiation dose at CT urography and conventional urography. Radiology 2004; 232(1):126–132. doi: 10.1148/radiol.2321030222 15220498 10.1148/radiol.2321030222

[2] Jinzaki M, Matsumoto K, Kikuchi E, Sato K, Horiguchi Y, Nishiwaki Y, et al Comparison of CT urography and excretory urography in the detection and localization of urothelial carcinoma of the upper urinary tract. AJR Am J Roentgenol 2011: 196(5):1102–1109. doi: 10.2214/AJR.10.5249 21512076 10.2214/AJR.10.5249

[3] Dillman JR, Caoili EM, Cohan RH. Multi-detector CT urography: a one-stop renal and urinary tract imaging modality. Abdom Imaging 2007; 32(4):519–529. doi: 10.1007/s00261-007-9185-5 17597338 10.1007/s00261-007-9185-5

[4] Caoili EM, Cohan RH, Korobkin M, Platt JF, Francis IR, Faerber GJ, et al Urinary tract abnormalities: initial experience with multi-detector row CT urography. Radiology 2002; 222:353–360. doi: 10.1148/radiol.2222010667 11818599 10.1148/radiol.2222010667

[5] Van Der Molen AJ, Cowan NC, Mueller-Lisse UG, Nolte-Ernsting CCA, Takahashi S, Cohan RH, et al CT urography: definition, indications and techniques. A guideline for clinical practice. Eur Radiol 2008; 18(1):4–17. doi: 10.1007/s00330-007-0792-x 17973110 10.1007/s00330-007-0792-x

[6] Silverman SG, Akbar SA, Morlele KJ, Tuncali K, Bhagwat JG, Seifler JL. Multi-Detector Row CT Urography of Normal Urinary Collecting System: Furosemide versus Saline as Adjunct to Contrast Medium. Radiology 2006; 240(3):749–755. doi: 10.1148/radiol.2403050233 16926326 10.1148/radiol.2403050233

[7] Caloili EM, Cohan RH, Korobkin M. Effectiveness of abdominal compression during renal helical CT. Acad Radiol 2001; 8(11):1100–1106. doi: 10.1016/S1076-6332(03)80721-3 11721809 10.1016/S1076-6332(03)80721-3

[8] Meindl T, Coppenrath E, Kahlil R, Muller-Lisse UL, Reiser MF, Muller-Lisse UG. MDCT urography: retrospective determination of optimal delay time after intravenous contrast administration. Eur Radiol 2006; 16(8):1667–1674. doi: 10.1007/s00330-006-0149-x 16583216 10.1007/s00330-006-0149-x

[9] Meindl T, Coppenrath E, Degenhart C, Muller-Lisse UL, Reiser MF, Muller-Lisse UG. MDCT urography: experience with a bi-phasic excretory phase examination protocol. Eur Radiol 2007;17(10): 2512–2518 doi: 10.1007/s00330-007-0600-7 17429641 10.1007/s00330-007-0600-7

[10] Caoili EM, Inampudi P, Cohan RH, Ellis JH. Optimization of multi-detector row CT urography: effect of compression, saline administration and prolongation of acquisition delay. Radiology 2005; 235(1):116–123. doi: 10.1148/radiol.2351031085 15716388 10.1148/radiol.2351031085

[11] Dillman JR, Caoili EM, Cohan RH, Ellis JH, Francis IR, Nan B, et al Comparison of Urinary Tract Distension and Opacification Using Single-Bolus 3-Phase vs Split-Bolus 2-Phase Multidetector Row CT Urography. J Comput Assist Tomography 2007; 31:750–757. 10.1097/RCT.0b013e318033df36 17895787

[12] Kim S, Wang LL, Heiken JP, Siegel CL, Hildebolt CF, Bae KT. Opacification of Urinary Bladder and Ureter at CT Urography: Effect of a Log-rolling Procedure and Postvoiding Residual Bladder Urine Volume. Radiology 2008; 247(3):747–753. doi: 10.1148/radiol.2473070965 18413887 10.1148/radiol.2473070965

[13] Honda O, Yanagawa M, Inoue A, Kikuyama A, Yoshida A, Sumikawa H, et al Image quality of multiplanar reconstruction of pulmonary CT scans using adaptive statistical iterative reconstruction. Br J Radiol 2011; 84(1000):335–341. doi: 10.1259/bjr/57998586 21081572 10.1259/bjr/57998586 PMC3473477

[14] Sagara Y, Hara AK, Pavlicek W, Silva AC, Paden RG, Wu Q. Abdominal CT: Comparison of low-dose CT with adaptive statistical iterative reconstruction and routine-dose CT with filtered back projection in 53 Patients. AJR Am J Roentgenol 2010; 195(3):713–719. doi: 10.2214/AJR.09.2989 20729451 10.2214/AJR.09.2989

[15] Juri H, Matsuki M, Inada Y, Tsuboyama T, Kumano S, Haruhito A, et al: Low-dose computed tomographic urography using adaptive iterative dose reduction 3-dimensional: Comparison with routine-dose computed tomography with filtered back projection. J Comput Assist Tomography 2013; 37(3):426–431. 10.1097/RCT.0b013e3182830aa9 23674016

[16] Metser U, Goldstein MA, Chawla TP, Fleshner NE, Jacks LM, O’Malley ME. Detection of Urothelial Tumors: Comparison of Urothelial Phase with Excretory Phase CT Urography- A Prospective study. Radiology 2012; 264(1):110–118. doi: 10.1148/radiol.12111623 22495683 10.1148/radiol.12111623

[17] Hack K, Pinto PA, and Gollub MJ. Targeted Delayed Scanning at CT Urography: A Worthwhile Use of Radiation? Radiology 2012; 265:143–150. doi: 10.1148/radiol.12110548 22855323 10.1148/radiol.12110548

